# Catalysing progressive uptake of newer diagnostics by health care providers through outreach and education in four major cities of India

**DOI:** 10.1371/journal.pone.0193341

**Published:** 2018-03-06

**Authors:** Neeraj Raizada, Sunil D. Khaparde, Soumya Swaminathan, Sanjay Sarin, Virender Singh Salhotra, Aakshi Kalra, Ashwani Khanna, K. K. Chopra, M. Hanif, K. R. Umadevi, Syed Hissar, Sreenivas Achuthan Nair, C. H. Surya Prakash, B. K. Saha, Raghuram Rao, Claudia Denkinger, Catharina Boehme

**Affiliations:** 1 Foundation for Innovative New Diagnostics, New Delhi, India; 2 Central TB Division, Government of India, New Delhi, India; 3 Indian Council of Medical Research, New Delhi, India; 4 State TB office, Government of NCT, New Delhi, India; 5 New Delhi TB Centre, New Delhi, India; 6 National Institute for research in Tuberculosis, Chennai, India; 7 World Health Organization, Country Office for India, New Delhi, India; 8 Intermediate Reference Laboratory, Hyderabad, India; 9 Intermediate Reference Laboratory, Kolkata, India; 10 Foundation for Innovative New Diagnostics, Geneva, Switzerland; Indian Institute of Technology Delhi, INDIA

## Abstract

**Background:**

Unlike in adults, diagnosis of TB can be challenging in children, as signs and symptoms of paediatric TB can be very non-specific and similar to other common childhood chest infections, which may lead to under or delayed diagnosis of TB disease. In spite of the increasing availability of rapid high-sensitivity diagnostics in public and private sectors, majority of paediatric TB cases are empirically diagnosed, without laboratory confirmation. To address these diagnostic challenges, World Health Organization (WHO) has recommended upfront Xpert MTB/RIF (Xpert) testing for the diagnosis of TB in paediatric presumptive pulmonary and extra-pulmonary TB (EPTB) cases. However, in spite of the increasing availability of rapid high-sensitivity diagnostics, a significant gap exists in its application with Xpert being rarely used as an upfront diagnostic among patients presumed to have TB. Under an ongoing paediatric project since April 2014, which provided free-of-cost upfront Xpert testing, several low-cost outreach and education interventions were undertaken to increase the diagnostic uptake by different providers catering to the paediatric population, thereby increasing adherence to global guidance.

**Methods:**

Providers catering to paediatric population in the project cities were systematically mapped and contacted using different outreach strategies. The focus of outreach efforts was to increase provider literacy and increase their awareness of the availability of free rapid diagnostic services with the goal of changing their diagnostic approaches.

**Results:**

From April 2014 to June 2016, more than 5,700 providers/facilities were mapped and 3,670 of them were approached. The number of providers/facilities engaged under the project increased more than 10-fold (43 in April, 2014 to 466 in June, 2016), with significant increase in project uptake, both from public and private sector. Overall 42,238 paediatric presumptive TB cases were enrolled in the project, across the four cities. Over the project period, quarterly diagnostic uptake and paediatric TB cases detection rates increased more than two-fold. TB detection rates were similar in patients from public and private sectors.

**Conclusions:**

Ongoing efforts in scaling up new rapid diagnostics involves significant investments. These efforts need to be complemented with proactive provider engagement to ensure provider-literacy and awareness, for maximizing impact of this scale-up. The current project demonstrated the usefulness of outreach and education interventions for the effective uptake of newer diagnostics.

## Introduction

Globally, under-diagnosis of childhood tuberculosis remains an obstacle to effective management [1,2]. In high TB burden settings, it is estimated that childhood TB contributes to 15–20% of all TB cases and is one of the leading cause of childhood mortality [2]. India is the highest TB and DR-TB burden country globally, and in 2016, 76,475 paediatric TB cases were notified accounting for 5% of notified TB cases in India [3]. Immature immune system and the poor performance of traditional microbiological tests in children poses challenges for bacteriological confirmation of TB disease in children [1, 4]. Unlike in adults, clinical diagnosis of TB can be challenging in children, as signs and symptoms of TB in children can be very non-specific and similar to other common childhood chest infections [1], leading to possibility of under or delayed TB diagnosis.

The Xpert assay, a sensitive and specific diagnostic tool, offers a promising solution to achieve the global objective of early and accurate detection of TB and rifampicin-resistant TB which is crucial for the timely initiation of accurate treatment in this vulnerable population [5,6]. To address these diagnostic challenges, WHO has recommended upfront Xpert MTB/RIF (Xpert) testing for the diagnosis of TB in paediatric presumptive pulmonary and EPTB cases [7,8]. However, in spite of the increasing availability of Xpert in public and private sectors, a significant gap exists in its application with Xpert rarely being used as an upfront diagnostic among patients with presumed TB [9–11]. As a result, the majority of paediatric TB cases are clinically diagnosed, without laboratory confirmation [12,13].

Against this backdrop, the current project was undertaken in four major cities of India, (Delhi, Chennai, Hyderabad and Kolkata) from April 2014. The project aimed to catalyze adoption of upfront Xpert testing in paediatric presumptive TB cases as routine practice by providers. We report here our experience in engaging various providers in these four cities for the uptake of the improved diagnostic strategy in public and private sectors from April 2014 to June 2016.

## Methods

The current project was implemented in four major cities of India, namely Chennai, Delhi, Hyderabad and Kolkata, having a total population over 30 million. Systematic outreach and education initiatives were undertaken in the project, with clearly defined project steps—mapping, approaching, engagement and follow-up of health care providers. Mapped providers were methodically ‘approached’- i.e. reached out though different outreach and education strategies for engagement, i.e. referral of specimen of paediatric presumptive TB cases for Xpert testing, under the project, and subsequently followed-up regularly.

Providers catering to the healthcare needs of the paediatric population were systematically mapped and several approaches were adopted to effectively map providers relevant to the project, on an ongoing basis, since the outset. These included snow-balling, key informant and patient interview, browsing of web based data bases of providers, liaising with chemists and medical representatives of pharmaceutical companies, etc. Other mapping approaches adopted also included liaising with professional associations of paediatricians and chest physicians to get access to the data bases of professionals registered with them.

The mapped providers were systematically approached through several interventions namely, distribution of project flyers and brochures, one-on-one meetings, telephonically and through invitation to Continuing Medical Education (CME) events organised under the project. Flyers and brochures were distributed, which were periodically updated and couriered to mapped providers. Flyer distribution was followed up by telephonic contact with the providers and where feasible one-on-one meeting by the project team. Flyers were also handed over to providers when they were approached through one on one meetings. While the project flyers ensured broader coverage, one-on-one meetings with providers had a more personalised approach in establishing a rapport and leading to better engagement. These efforts were complimented by periodic comprehensive CME events which provided update on topics relevant to the project interventions including WHO guidelines on use of Xpert and treatment aspects which were covered by key speakers. Such events encompassed engagement with various health providers in public and private sector for participation under the current project.

A provider, who had once sent a patient sample of a paediatric presumptive TB case to the project lab, was considered as ‘engaged’. These engaged providers were followed up at regular intervals, for feedback and to sustain the linkage.

To ensure effective and increasing uptake of project interventions, several of these outreach and education approaches were undertaken simultaneously on regular basis. The provider database was maintained in each of the four project cities, with details of mapped, approached and engaged providers. This database was kept dynamic and updated on a day-today basis. For the project monitoring purposes, facility with single or multiple health care providers was taken as single unit, for the purpose of mapping, approaching and engagement, e.g. a paediatric department of a tertiary care facility was considered as a single unit; similarly, a primary health care centre with a single health care professional or a standalone clinic of a paediatrician was also considered a single unit.

### Project details

The services provided under the project included free-of-cost upfront Xpert testing for all presumptive paediatric TB patients, through a dedicated high through-put Xpert laboratory linked with different public and private sector providers across the city through specimen transportation linkages, implementing a hub and spoke model. Different providers (both public and private) in the project cities were given an option of prescribing free of cost Xpert testing to the paediatric presumptive TB cases identified by them in their respective health facilities. The health care providers were requested to refer the different types of patient specimen, using rapid specimen transportation linkages for Xpert testing. Specimen transportation linkages were optimised and a rapid reporting mechanism was established to ensure that the test results were communicated to all health providers and referring facilities by e-mail and short messaging service (SMS) within 24 hours of specimen collection. To facilitate incremental project uptake, engagement under the project was simplified, and any provider/facility in the city could refer specimen/s of paediatric presumptive TB case by filling up a standard RNTCP form with a) patient details and b) provider contact details, to avail free testing for their patients. No separate procedure/ process was required for engagement under the project. A dedicated lab team was hired under the project to ensure same day testing and effective communication with all provider. Since Xpert testing is automated, requiring minimal manual processing, the lab team also spearheaded the project outreach and education activities.

### Data management

Data for all the approached and engaged providers was recorded systematically after each outreach and education intervention. Microsoft Excel 2013 was used for cleaning the data and data was analysed using R programming. All confidence intervals were calculated based on the binomial distribution with 95% probability interval (S1 Data).

For analytical purpose, engaged providers were classified based on the quantum of referrals as high, moderate and low quantum referring provider, based on their workload. The providers with average monthly referrals of more than 10 patients’ samples, were defined as high quantum referring providers. Similarly, providers with monthly average of more than 5 and less than 10, as moderate and providers with less than 5 patient samples as moderate and low quantum referring providers, respectively. All providers who referred from a single facility/hospital were clubbed together as a single unit.

All costs were evaluated and expressed in 2015 U.S. dollars where cost data items in local currencies were converted to the US dollar according to the average UN operational exchange rate in 2015 [14]

### Ethical issues

Xpert testing for paediatric presumptive TB cases is an approved intervention under RNTCP. The current project was undertaken by FIND, after approval from and in collaboration with RNTCP. As such, the results presented here are our experience-sharing of implementing approved interventions in a programmatic setting within the existing accredited RNTCP TB diagnostic lab network. Since the observations described here are a part of implementation of approved interventions under RNTCP and a part of Standard of TB care in India, separate ethical clearance was not required.

## Results

Starting April, 2014 to June, 2016, a total of 5,738 providers catering to paediatric populations or paediatric presumptive TB cases were mapped in the four project cities. Of these, 4,494 (78.3%) were private sector providers while the rest 21.7% were public sector providers. Since mapping was an ongoing activity and substantial number of providers were either so far not approached or were in the process of being approached, at the point of data analysis details of their educational background was not available. During nine quarters of implementation, overall 42,238 paediatric presumptive TB cases were enrolled under the project, and 3,340 (7.9%, CI 7.7–8.2) paediatric TB cases were diagnosed, of which, 295 (8.8%; CI 7.9–9.9) were rifampicin-resistant TB (DR-TB) cases.

At the point of data analysis, of the mapped providers, a total of 3,670 (64.0% CI: 62.7–65.2) were approached by the project team by various outreach and education intervention, most being approached by multiple interventions (Table 1). Majority of the approached providers were periodically mailed printed standardised literature on the project and project updates, by means of project flyers and brochures. The project team had one on one meetings with 2023 (35.3% CI: 34.0–36.5) mapped providers, to sensitize them about the project, and/or to follow-up with them and provided project updates with the objective of effective engagement under the project. Telephonically, 1323 (23.1% CI: 22.0–24.1) providers were approached. In addition, during the project duration a total of 33 Continuing Medical Education events were organised in the 4 project cities. Through these CMEs a total of 1906 (33.2% CI: 32.0–34.5) providers were approached. Since multi-prong approach was used in the project to sensitize the providers therefore these figures presented here are overlapping figures.

**Table 1:** Providers approached using multiple outreach and education intervention.

S. No.	Outreach and education intervention		Approached providers
1.	Flyers		1589
2.	One on One meeting/Flyers		758
3.	One on One meeting/Telephonically		559
4.	One on One meeting/Telephonically/Flyers		706
5.	Telephonically/Flyers		58
	**Grand Total**		**3670**

Overall direct expenses incurred for the mailing of project literature and updates was USD 505.0, having one-on-one meetings with providers was USD 1,210.2, and the cost incurred for telephonically contacting and following up with different providers was USD 28.8. A number of providers were approached by a combination of these interventions for which combined expense of USD 1579.6 was incurred. However, this figure does not include cost for Delhi site. Together, these interventions costed USD 3,323.7 over the 9 project quarters. Over and above these expenses, a total of 33 CMEs were organised which together costed USD 11,365.80, bringing the total direct costs of these outreach and education approaches to USD 14,689.50. Since many of the mapped providers were approached by multiple methods, and there were multiple follow-ups, over the 9 quarters of the project, exact cost calculation per intervention per provider could not be ascertained.

**Fig 1:**
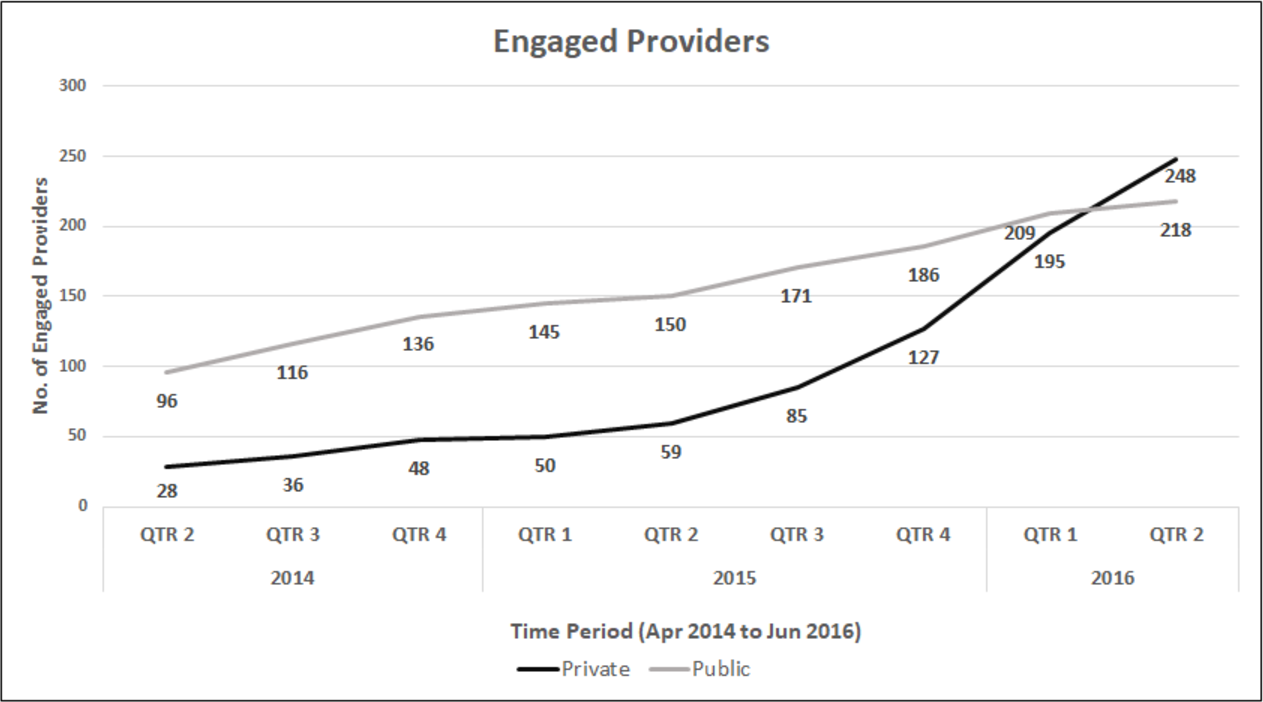
Engaged providers under the project.

**Fig 2:**
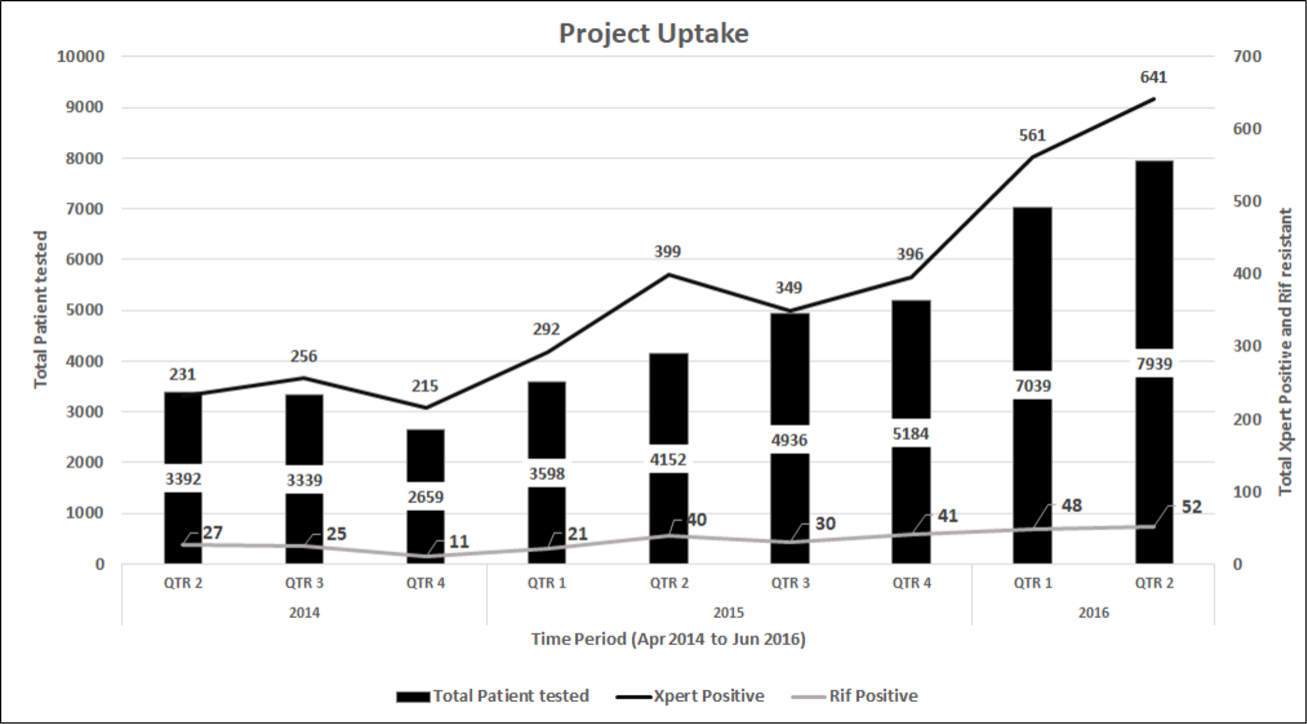
Paediatric project uptake over every successive quarter during the reported period.

As a result of various outreach and education interventions to catalyse progressive uptake of rapid diagnostic testing for paediatric TB, we observed an overall increasing trend, both in number of providers engaged and project uptake every successive quarter. The number of providers engaged under the project increased from 124 (96 public sector providers and 28 private sector providers) in the first quarter (Q1) of the project to 466 (218 public sector provider and 248 private sector providers) in the 9^th^ quarter (Q9) of the project. During the initial quarters of the project, proportion of providers was heavily dominated by public sector providers, with a steep incremental trend in the number of new public sector providers engaging every quarter. In the same initial quarters of the project, engagement of new private sector providers showed in comparison a static trend. Starting the fifth quarter of the project, steep increasing trends were observed in the number of new private providers engaging in the project, with an approximate 50% increase every successive quarter; leading to a reversal in proportions by ninth project quarter, in favour of more private providers. (Fig 1) Incremental project uptake was observed every successive quarter, increasing from 3,392 paediatric presumptive TB cases being enrolled in Q1 of the project to 7,939 in Q9 of the project. The number of TB cases detected increased from 231 (6.8% CI: 6.0–7.7) in Q1, of which 27 were rifampicin resistant cases to 641 (8.1% CI: 7.5–8.7) in Q9 of which 52 were rifampicin resistant. (Fig 2)

Of the 466 providers engaged under the project, 200 (42.9% CI: 38.5–47.5) were paediatricians, 180 (38.6% CI: 34.3–43.1) were general physicians, 67 (14.4% CI: 11.5–17.9) were chest specialist. Majority of the engaged paediatricians were from private sector (149/200; 74.5% CI: 68.0–80.0) and majority of the engaged chest specialists were from the public sector (59/67; 88.1% CI: 78.2–93.8). Similar proportion of engaged general physicians were from public and private sector (94 (52.2% CI: 45.0–59.4) vs. 86 (47.8% CI: 40.6–55.0)).

Of the 42,238 paediatric presumptive TB cases enrolled in the project, 25,535 (60.5% CI: 60.0–60.9) were referred by paediatricians, 5,009 (11.9% CI: 11.6–12.2) by chest specialists, 3,910 (9.3% CI: 9.0–9.5) by general physicians and 7,784 (18.4% CI: 18.1–18.8) by medical providers of other specialities. In the enrolled presumptive TB cases enrolled, 3,340 (7.9%, CI 7.7–8.2) paediatric TB cases were diagnosed, of which, 295 (8.8%; CI 7.9–9.9) were rifampicin resistant TB (DR-TB) cases. Of the detected TB cases, majority of the TB cases were detected amongst referrals from paediatricians and chest specialists, 2,150 (64.4% CI: 62.7–66.0) and 709 (21.2% CI: 19.9–22.6), respectively.

Of the engaged providers, 43 (9.2% CI: 6.9–12.2) were high quantum referring providers. Overall, the high referring providers, contributed 34,852 of the 42,238 presumptive paediatric TB cases (82.5% CI: 82.1–82.9), of which 2619 (7.5% CI: 7.2–7.8) were found positive for TB on Xpert. Amongst the high referring providers, no significant variation in TB positivity was observed between public and private sector (2477/ 32714; (7.6% CI: 7.3–7.9) vs. 142/2138; (6.6% CI: 5.7–7.8)). Further, a total of 35 (7.5% CI: 5.4–10.3) providers were identified as moderate providers which contributed 3,442 (8.1% CI: 7.9–8.4) of paediatric presumptive TB referrals, detecting 251 (7.3% CI: 6.5–8.2) positive TB cases on Xpert. Amongst these providers, the TB positivity was significantly higher in referrals from public sector as compared to private sector (210/2572; 8.2% CI: 7.2–9.3 vs. 41/870; 4.7% CI:3.5–6.3). Low quantum referring providers were 388 (83.3% CI: 79.6–86.4). These contributed 3,944 (9.3% CI: 9.1–9.6) of the presumptive TB cases enrolled under the project, of which 470 (11.9% CI: 10.9–13.0) were found TB positive on Xpert, with similar TB positivity in referrals from public and private sector (347/2761; 12.6% CI: 11.4–13.9 vs 123/1183; 10.4% CI: 8.8–12.3, respectively) (Table 2). The high quantum referring providers contributed, 78.4% (CI: 77.0–79.8) of the detected paediatric TB cases.

**Table 2:** Engaged providers under the project stratified by quantum of referrals and type of providers.

S. No.	Engaged providers	n	Type of provider	
			Public	Private
1.	High quantum referring providers	43	37	6
2.	Moderate quantum referring providers	35	29	6
3.	Low quantum referring providers	388	152	236
	**Grand Total**	**466**	**218**	**248**

Overall the public sector providers contributed 38,047 (90.1% CI: 89.8–90.4) and private sector providers contributed, 4191 (9.9% CI: 9.6–10.2) of the referrals. TB positivity rates were similar in referrals from public and private sector (8.0% CI: 7.7–8.3 vs. 7.3% CI: 6.6–8.1, respectively). No significant gender variation or age variation was observed amongst referral from public and private sector.

Over the 9 quarters of project implementation, we were able to extend the project interventions to 42,238 children, detecting 3340 paediatric TB cases of which 295 were DR-TB cases. We were able to reach-out to 3,670 providers and engage 466 providers. The expenses incurred to catalyse incremental project i.e. USD 14,689.5, translated to USD 0.3 per presumptive TB case tested, 4.4 per paediatric TB case detected and 49.8 per paediatric rifampicin resistant TB case detected. This also translated to USD 4.0 per approached provider and USD 31.5 per engaged provider. The average expense on various outreach and education intervention translated to USD 1,632.1 per quarter. The direct cost incurred on the outreach and education activities, not including human resources, represented 1.5% CI: 1.5–1.6 of the overall project cost.

## Discussion

Globally, there remains a major gap between the estimated burden and notification of paediatric TB cases [11,15–21]. In spite of the major recent progresses in the development of TB diagnostics and available evidences specifically in context of Paediatric TB and EP-TB, underdiagnoses of paediatric TB remains a major obstacle in its effective management [6, 22–24]. WHO has issued guidelines which have been adopted by RNTCP, to address these TB diagnostic challenges in children. These guidelines recommended providing paediatric presumptive TB cases upfront access to rapid, high sensitivity TB diagnostic assay, Xpert [7,8]. Role of advocacy with various providers for the better uptake of TB control services has been flagged in various forums [25–27]. The current project was undertaken to catalyse the implementation of these guidelines in public and private sector, by improving provider literacy, providing free of cost access to Xpert testing in four major cities of India, and simplifying processes involved in availing this free intervention. This initiative is one of largest global efforts, exclusively dedicated to the implementation of WHO guidelines on the use of upfront Xpert testing for paediatric population.

Outreach and education efforts to catalyse the incremental uptake of Xpert testing for paediatric presumptive TB cases led to an increasing quarterly trend in number of children being enrolled, with the quarterly quantum of enrolment increasing more than two fold over the nine project quarters. Since the project outset, systematic outreach and education approaches to provider engagement were undertaken using a spectrum of high coverage and intensive contact interventions, which led to incremental project uptake. Earlier studies have documented the role of outreach and education in increasing the uptake of TB control interventions [26,27]. Simple low cost outreach and education approaches, at one hand ensured utilisation of existing project laboratory staff for Xpert testing being involved in outreach and education activities, and on the other hand replicability of these approaches, beyond project duration and in other settings. Xpert, an automated, rapid test offering upfront results for TB and susceptibility for rifampicin, when applied to different types of specimen from paediatric presumptive TB cases, under uncontrolled field conditions led to incremental detection of TB and quite remarkably large number of rifampicin resistant TB cases. These findings disseminated through the different outreach and education channels further reinforced the utility of the project interventions with approached providers. Overall the increasing trend in the uptake of project interventions and provider engagement reported here demonstrate the utility of different outreach and education approaches and make a case for replicating this low cost outreach and education model piloted here in other settings.

Sectoral comparison (public vs. private) of the trends in provider engagement over time showed contrasting results. Initial project quarters saw a provider engagement predominantly from public sector. Increasing trends in engagement of private providers were observed starting 5^th^ quarter of the project, and by the 9^th^ quarter, a reversal in the proportion was observed, i.e. higher proportions of private providers engaged in the project as compared to public sector providers. These trends were observed in spite of no sectoral variation in outreach and education efforts. These findings could partly be explained by initial reluctance in the private sector in engaging in a new initiative, which was largely addressed when the initial results were available and widely disseminated [23]. Further, several initial procedural teething bottle-necks in the interfacing of the two sectors, where in a private provider is empowered to prescribe a free test in lab located in the public sector, were systematically identified and addressed, thereby streamlining engagement process. Quite significantly, as revealed during interaction with the providers, the initial results showed high levels of rifampicin resistance in the diagnosed paediatric TB cases under the project [23]. These results led to a need for microbiological confirmation of TB diagnosis along with upfront drug susceptibility testing amongst the treating providers, leading to increased willingness to engage amongst the providers.

Results from the current pilot provide some useful unique insights into the paediatric TB care pathways, and largely mirror the Pareto principle [28]. It was observed that high quantum referring providers/facilities, constituted less than 10 percent of the engaged providers and contributed > 80% of the enrolled presumptive TB cases. Most of these facilities were tertiary care facilities, and majority were public sector facilities. Providers with low quantum of referrals, which comprised > 80% of the engaged providers had large number of providers from private sector. While this group of providers contributed around 10% referrals, TB positivity in referral was significantly higher as compared to providers with higher quantum of referrals. This group of providers comprised of either individual community based private clinics or primary health care facilities, which invariably represented the first point of contact for health seeking. Engagement with these providers was found to be useful from the perspective of project penetration and minimizing patient pathway delays as documented by previous study in one of the project settings [29]. It was further observed that in spite of approaching a large number of providers, majority of paediatric TB cases detected under the project were contributed by paediatricians, followed by chest specialists. Review of literature did not provide useful insights into trends observed by other researchers in paediatric patient pathways and project data limitations make it challenging to comment of whether these providers were the first point of contact for paediatric presumptive TB cases. However, these findings might be useful for designing specific interventions for paediatric TB.

In spite of similar outreach and education efforts, both in public and private sector, referrals from private sector constituted less than 10% of the project cohort. TB positivity rates, in private sector referrals were similar to public sector. Similar findings have been reported in earlier studies [30].This lower representation of private sector referrals in the project cohort, could in part be attributed to increased momentum in private sector engagement observed only after fifth quarter of the project. This could also be due to the project’s inability in engaging several private tertiary care institutions. The keys reasons identified for non-engagement of private sector tertiary care facilities under the current project was either availability of Xpert testing within some of these facilities or existing tie up with a private laboratory service provider/s. As such linkage to free of cost testing at the project Xpert labs from these facilities could not be achieved in several private tertiary care institutions. After outreach and education efforts in these facilities, providers may have prescribed upfront Xpert testing for paediatric presumptive TB cases at private laboratories; however the same is not captured under the data presented here. It may be worth flagging here, that while the overall contribution of private sector was significantly less than public sector, we observed similar incremental trends in referrals from private sector, which however, were overshadowed by the scale of cases from the public sector.

Ongoing efforts in scaling up new rapid diagnostics for tuberculosis involves significant investments in the health systems [31–34]. Major threat which may undermine the impact of these investments is sub-optimal uptake and low test volumes have already been reported from several settings globally [33,35,36]. These efforts need to be complemented with proactive outreach and education efforts to ensure provider engagement to ensure provider-literacy and awareness, for maximizing impact of this scale-up. Current project, with low cost outreach and education interventions, was successful in achieving an incremental project uptake and increasing implementation of global and national guidelines for the utilisation of Xpert in paediatric TB care. The project demonstrated the usefulness complementing similar new initiatives with outreach and education interventions for the effective uptake, without substantial additional expenses. While, the simplified nature of the advocacy interventions, made it feasible for implementation by existing laboratory staff, it also ensured the feasibility of replicating the current implementation model in other settings using existing field staff.

## Conclusion

The current project demonstrated the usefulness of complementing new diagnostic initiatives with outreach and education interventions aimed at provider behaviour change. Allocating a small proportion of project budget and project staff time to simple advocacy interventions led to incremental uptake of project intervention. The current project provided useful insights into the paediatric patient pathways and was able to engage an increase number of providers from public and private sector.

## Supporting information

S1 Data Compiled Sheet)_providers (Approached&Engaged).xlsb. (XLSB)
